# Interventions targeting patients with co-occuring severe mental illness and substance use (dual diagnosis) in general practice settings – a scoping review of the literature

**DOI:** 10.1186/s12875-024-02504-3

**Published:** 2024-08-03

**Authors:** Katrine Tranberg, Bawan Colnadar, Maria Haahr Nielsen, Carsten Hjorthøj, Anne Møller

**Affiliations:** 1 The Section of General Practice and the Research Unit for General Practice, Department of Public Health, University of Copenhagen, Copenhagen, Denmark; 2 Mental Health Center Copenhagen, Copenhagen Research Center for Mental Health – CORE, Copenhagen University Hospital, Copenhagen, Denmark; 3 Section of Epidemiology, Department of Public Health, University of Copenhagen, Copenhagen, Denmark

**Keywords:** Dual diagnosis, Co-occurring mental and substance use disorder, General practice, Scoping review, Intervention research, Primary care, Family medicine, Co-occurring disorders

## Abstract

**Background:**

People with dual diagnosis die prematurely compared to the general population, and general practice might serve as a setting in the healthcare system to mend this gap in health inequity. However, little is known about which interventions that have been tested in this setting.

**Aim:**

To scope the literature on interventions targeting patients with dual diagnosis in a general practice setting, the outcomes used, and the findings.

**Design and setting:**

A scoping review of patients with dual diagnosis in general practice.

**Methods:**

From a predeveloped search string, we used PubMed (Medline), PsychInfo, and Embase to identify scientific articles on interventions. Studies were excluded if they did not evaluate an intervention, if patients were under 18 years of age, and if not published in English. Duplicates were removed and all articles were initially screened by title and abstract and subsequent fulltext were read by two authors. Conflicts were discussed within the author group. A summative synthesis of the findings was performed to present the results.

**Results:**

Seven articles were included in the analysis. Most studies investigated integrated care models between behavioural treatment and primary care, and a single study investigated the delivery of Cognitive Behavioral treatment (CBT). Outcomes were changes in mental illness scores and substance or alcohol use, treatment utilization, and implementation of the intervention in question. No studies revealed significant outcomes for patients with dual diagnosis.

**Conclusion:**

Few intervention studies targeting patients with dual diagnosis exist in general practice. This calls for further investigation of the possibilities of implementing interventions targeting this patient group in general practice.

**Supplementary Information:**

The online version contains supplementary material available at 10.1186/s12875-024-02504-3.

## Introduction

It is well known that patients with severe mental illness (SMI) die up to 10–20 years before the general population [1]. Moreover, studies have shown that when mental illness is accompanied by a substance use disorder (SUD), the mortality gap increases further [2, 3]. This simultaneous presence of both severe mental illness and SUD is referred to as dual diagnosis or co-occurring disorders [4] and occurs in approximately 30% of all patients diagnosed with SMI [5]. There is no official definition of dual diagnosis, and dual diagnosis is not coded in the international coding system (ICD-10), however, the overall understanding of the term is that patients are affected by at least one mental illness and substance abuse disorder at the same time [4].

Having a dual diagnosis is associated with several adverse outcomes such as a higher incidence of recurrence of mental illness [6], increased hospitalization and readmissions [7], infections such as HIV [8] and Hepatitis C [9], and increased mortality [2, 3]. The lower average life expectancy can, in addition to suicidal behaviour and overdose, be largely attributed to an increased risk of several chronic health conditions [3, 6, 10–14], with cardiovascular disorders reported as being the greatest risk [15]. Even though patients with a dual diagnosis evidently need treatment for somatic diseases, research shows that the quality of the treatment provided fluctuates. There are several examples of this patient group being disregarded for their physical health problems, or the patients’ physical symptoms being misinterpreted by the health personnel as mental symptoms [16, 17].

In line with increased awareness of the patient group, the high prevalence of SUD among patients with SMI, and the complex issues associated with the diagnosis, the primary healthcare contact points for these patients vary across healthcare systems and contexts. However, primary care generally serves as the patient’s first point of contact with the healthcare system. Because general practitioners (GPs) in most cases act as gatekeepers to the secondary system, and thus potentially facilitates early discovery of disorders, their role in the healthcare system is essential for referring patients and managing their care. General practice could play a pivotal role in coordinating treatment for patients with dual diagnosis, and furthermore the GP is important in the detection, assessment, treatment, follow-up, and continuity of treatment of somatic diseases – the primary cause of premature death in this patient group [17, 18]. From studies investigating the path or journey for people suffering from dual diagnosis in the healthcare system, we know that patients with dual diagnosis often fall between chairs [19]. Despite extensive research in the general field of dual diagnoses, less is known about the role of general practitioners in coordinating care, detecting diseases, or treating patients with dual diagnosis, and what interventions has been performed to improve the healthcare delivery in this setting. It is also unclear, which effect measures have been examined in evaluations of interventions, and whether they relate to contact, follow-up, or treatment goals [18, 20].

## Aim

The study investigated the literature on interventions in a general practice setting targeting patients with dual diagnosis (co-occurring severe mental illness and substance use disorder), and aimed to answer the following questions:

1. Which interventions targeting patients with dual diagnosis have been tested in a general practice setting?
2. Which outcomes did the studies use to evaluate the interventions, and what were the primary findings?

## Methods

This scoping review was assembled by the methods developed by Arksey and O’Malley [21]. This type of review is well suited to highlight significant gaps in the evidence [22]. No review protocol has been published previous to this study.

### Data sources, search strategy, and inclusion criteria

We used the electronic databases PubMed (Medline), PsychInfo, and Embase to identify scientific articles on interventions aimed at patients with co-occurrence of severe mental illness and substance use in general practice. The specific search string and strategy are shown in appendix 1. We defined severe mental illness as either a diagnosis with depression of moderate or severe degree, bipolar affective disorder, or schizophrenia. Substance use disorder was defined as any harmful use of either drugs or alcohol. Additionally, the article should evaluate an intervention or initiative linked to general practice or primary care. We excluded studies that included patients under 18 years of age, and studies that were not published in English. The search was not limited to study type, date of publication, or a specific country. A combination of MeSH terms, keywords, free text search, as well as related terms, were used with Boolean operators (AND, OR). Each search was adapted to the specific requirements of the individual database, where the search string was divided into blocks of dual diagnosis (block 1), general practice (block 2), and intervention (block 3). The block descriptions and the final search string can be found in Appendix 1. The search was performed on April 22nd, 2022 and an update was performed on February 1st 2024. We found no additional articles that fulfilled the inclusion criteria in the updated literature search.

### Study selection

The studies identified in the final literature search were initially exported to the Endnote reference database to remove duplicates [23]. Afterwards, we imported the references to Covidence screening program [24]. The initial article sample was screened twice by four researchers (BC read all abstracts, and all abstracts were also screened by one of the co-authors MN, KT, and AM). Articles were selected for full-text review, if they met the following criteria:

1) Patients with dual diagnosis (understood as co-occurring severe mental illness, as defined in the above section, and substance use of either drugs or alcohol), 2) Adults above 18 years, and 3) Article should include an evaluation of one or more interventions situated in general practice. Any conflicts regarding inclusion of articles during the initial screening process were discussed and resolved before moving on to full text screening. The full text screening was performed by BC and KT. Conflicts were discussed and resolved between all four researchers.

### Data extraction and synthesis

From the included studies, we extracted data on study design, participants, definition of dual diagnosis, and description of intervention content, outcome measures, and overall study findings described in the study. This task was performed by AM and KT and verified by the remaining authors. Afterwards, we described the design and content of the interventions in the included studies, and listed the outcome measures and overall study findings in a non-systematic narrative summary in the results section.

## Results

### Search and study selection

**Fig. 1:**
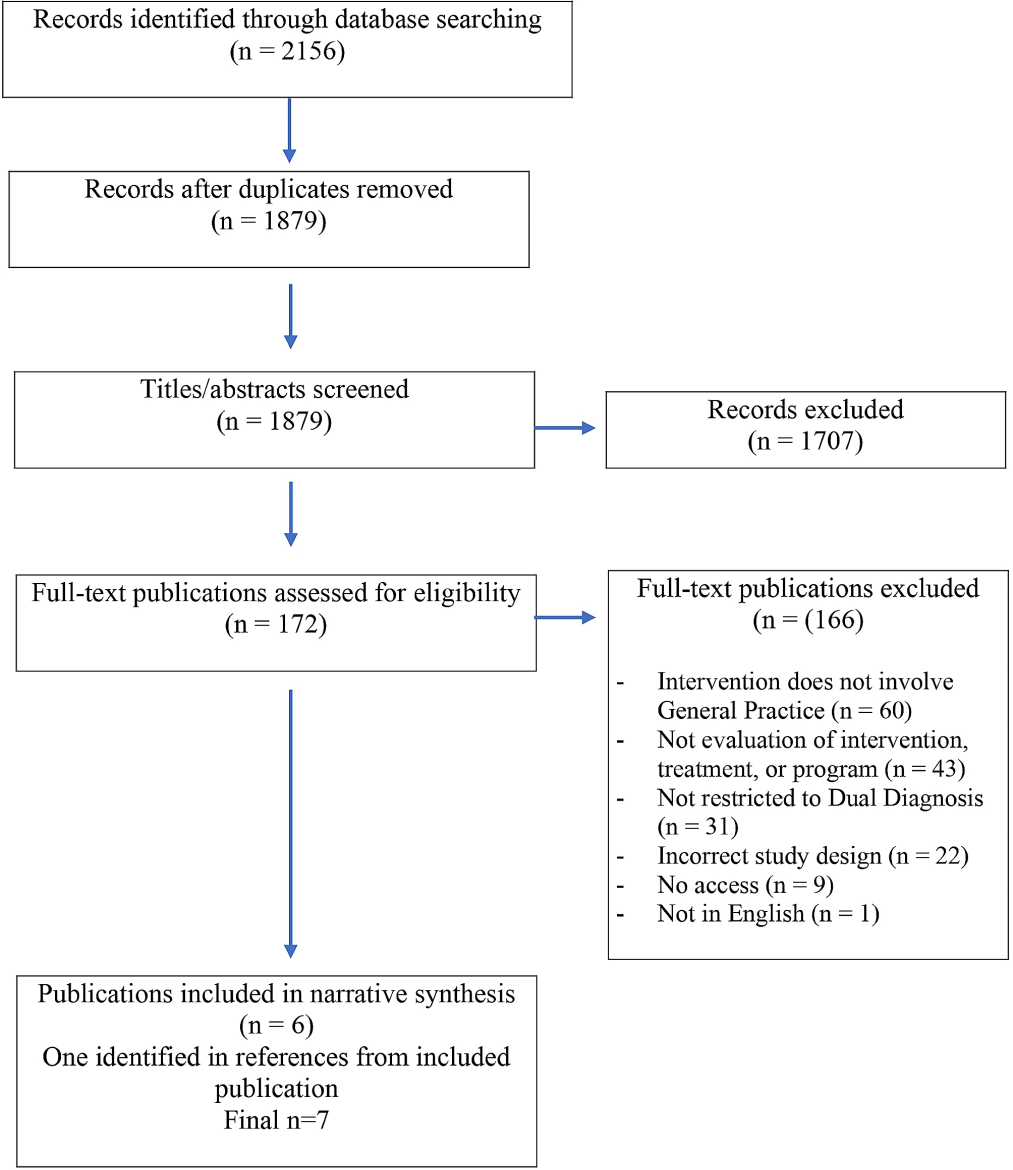
Study selection and exclusion process (PRISMA flow diagram)

The literature search in three databases yielded 1,879 articles after excluding duplicates. Our screening of both abstracts and titles, showed 172 articles were eligible for full-text review. Six articles met the final criteria and were included in the synthesis and one article was found in the reference list of one of the articles. The complete study selection and exclusion process is presented in Fig. 1. The general characteristics of the included articles are reported in Table 1. As described in Table 1, the studies from Oslin et al. and Bartels et al. origin from the same research project as well as the studies from Padwa et al. and Urada et al. All studies were conducted in the United States. In the updated literature search, we identified two studies, where it was unclear whether the intervention was based in general practice or a primary care setting. These studies were not included in the final synthesis.

**Table 1:** Overview of the included articles

	Study design, participants and setting	Definition of dual diagnosis	Intervention	Outcome measures	Findings
Park et al.	A post hoc sub group analysis from an RCT including participants with SUD and either major depressive disorder (n = 443) or PTSD (n = 205) participating in the AHEAD study. Participants were recruited from the residential detoxification unit in Boston, the Boston Medical Center (BMC), and through local advertisements. Exclusion criteria: Pregnancy, cognitive impairment, non-fluent in English or Spanish, or unable to provide contact information for tracking purposes. Enrollment of patients took place between September 2006 and 2008 and outcome assessments were performed three, six, and 12 months after enrollment.	**Major Depression and post traumatic stress disorder (PTSD)** Identified by using the Mini-International Neuropsychiatric Interview (MINI) **Alcohol or drug dependence (SUD)** Heavy alcohol use (consuming $4 standard drinks for women and $5 standard drinks for men at least twice or $15 drinks for women and $22 drinks for men in an average week) or drug use (psychostimulants or opioids) in the past 30 days assessed by the Composite International Diagnostic Interview–Short Form (CIDI-SF)	Patients were randomly assigned to either: **Chronic Care Management (CCM) ** Evidence-based treatments at the AHEAD clinic for substance dependence consisting of: clinical case management, motivational enhancement therapy, relapse prevention counseling, addiction pharmacotherapy, and referral to specialty addiction treatment and mutual-help groups. All elements were tailored to fit clinical needs and patient preferences. **Usual primary care** An appointment with a primary care physician at the BMC within approximately two to four weeks if the patient had no previous visits within the past three months and a list of addiction treatment resources.	**Alcohol or drug use** Use of any stimulants, opioids, or heavy drinking in the past 30 days measured by the Addiction Severity Index (ASI) and a 30-day timeline followback method. **Depressive symptom severity** Assessed with the PHQ-9 **Anxiety severity** measured with Beck Anxiety Inventory (BAI) **Consequences of alcohol and drug use** measured with Short Inventory of Problems for alcohol use (SIP-2R or SIP-alcohol) and a modified version of the SIP for drugs (SIP-drug) **Treatment utilization** emergency department visits (ED), hospitalizations, addiction treatment and mental health treatment.	**SUD and mental health outcomes** No significant differences were found between CCM and usual care. **Treatment utilization** CCM was not more effective than usual care in patients with depression or PTSD and co-occuring substance abuse regarding use of ED or hospitalisation or abuse or symptoms. A nearly significant effect was found regarding days at ED among patients with depression.
Bartels et al.	RCT study included primary care patients (n = 2.022) with co-occuring mental disorder and at-risk drinking, aged 65 years or older, recruited from 10 primary care and mental health units. Exclusion criteria: Diagnosis with psychosis, mania or hypomania; No previous enrollment in similar treatment. Patients were screened between March 2000 and October 2001 and followed over a six months period.	**Mental health disorder** Significant psychological distress on the General Health Questionnaire, and/or a positive response to suicidal ideation questions modified from PRIME-MD. **At-risk drinking** More than seven drinks/week or more than two binge episodes in the past three months consisting of more than three drinks on a single occasion.	Patients were randomly assigned to receive integrated care or enhanced referral care. **Integrated care** consisted of 1) Mental health and substance abuse services co-located in the primary care setting 2) Mental health and substance abuse services provided by licensed mental health/substance abuse providers 3) Communication on the clinical evaluation and treatment plan between the mental health and substance abuse clinician and primary care provider 4) An appointment with the mental health and substance abuse provider within two to four weeks following the primary care provider visit. **Enhanced referral care** consisted of 1) Referral within two- to four weeks of the primary care provider appointment 2) Treatment offered in a separate location by licensed mental health and substance abuse professionals 3) Agreement by the specialty mental health/substance abuse clinics to comply with model requirements, including time to first appointment and coordinated follow-up contacts if the patient failed to make the first scheduled visit 4) Assistance with transportation 5) Coverage for the costs of the specialty mental health and substance abuse visit.	**Treatment engagement** Defined as attendance at an appointment with a mental health/substance abuse provider after inclusion during the six months follow-up. For every encounter a form was filled by a clinician or research assistant.	**Treatment engagement** Integrated care showed a greater treatment engagement rate (71%) than enhanced referral (48.8%). Patients with dual diagnosis had more visits for the integrated model than enhanced care. Patients living far away from the treatment location showed a significantly decreased rate of treatment engagement. In total, older patients were more likely to accept integrated care within primary care than in a mental health/substance abuse clinic.
Oslin et al.	Same study as Bartels et al. A multisite RCT comparing integrated care with enhanced specialy referral. See Bartels et al for study population and exclusion criteria. Differs from Bartels et al. because only nine sites was included since the number of patients fulfilling criteria for at risk drinking was below 10. The study period was from March 2000 to March 2002. Outcomes were measured at three- and six months after enrollment	See Bartels et al.	See Bartels et al.	**Alcohol use** The quantity and frequency of alcohol use seven days before assessment at three and six months follow-up and the number of binge drinking episodes three months before each assessment.	**Alcohol use** The average quantity and frequency models show significant time effects with reduction in drinking by six months for all participants except for patients with a dual diagnosis. Binge drinking declined over time (but not after six months among patients with dual diagnosis). No significant difference was found between treatment groups. As reported by Bartels et al., the findings yielded minimal uptake and implementation of the interventions in both study groups and generally low participation in treatment
Kalapatapu et al.	A post hoc, sub group analysis from an RCT of primary care patients with major depressive disorder and problematic alcohol use (n = 103). Patients were above 18 years, able to speak and read English, and to participate in either face-to-face or telephone therapy. Patients were recruited from November 2007 to December 2010 and followed up to 18 weeks after baseline.	**Major depressive disorder** Hamilton Depression Rating Scale (Ham-D) score greater than or equal to 16. **Problematic alcohol use** Alcohol Use Disorders Identification Test (AUDIT) with a score of four or more for women, and for men a score of four or more above age 60, and a score of eight or more for men younger than 60 years of age.	Patients were randomly assigned (1:1) to: **Face-to-face cognitive-behavioral therapy (CBT)** Eighteen 45-minute sessions were provided to the patients with two weekly sessions in the first two weeks, followed by 12 weekly sessions, with two final booster sessions the last four weeks. Patients received a workbook covering the aspects of CBT concepts. PhD level psychologists provided the CBT treatment in a Preventive Medicine Clinic at Northwestern University. **Telephone-administered CBT (T-CBT)** Treatment was exactly the same as regular CBT except from being administered by telephone.	**Treatment adherence** (1) Number of CBT sessions attended; (2) Failure to engage in treatment (attended fewer than five sessions); (3) Failure to complete treatment (attended more than four CBT sessions but less than 18 CBT sessions); and (4) Discontinuation of treatment **Depression** (1) PHQ-9 Score; (2) 17-item Ham-D score; (3) Whether a participant was receiving an active dose of antidepressant medication; (4) Whether a participant continued to meet the criteria for major depressive disorder; (5) Response criteria on the Ham-D, defined as a 50% decrease in Ham-D score; and (6) Remission criteria on the seven-item Ham-D, defined as less than or equal to seven. **Alcohol use disorder** (1) AUDIT score	**Treatment adherence** No significant differences were detected between groups on any outcomes. In total 24.5% in the CBT group and 26% in the T-CBT group discontinued treatment. The sub-analysis showed that participants who discontinued treatment were more likely to be single and meet remission criteria on the HAM-D score. **Depression** No differences were detected between the CBT and T-CBT group on the 17-item HAM-D score at neither end of treatment nor at three- and six months follow-up. No statistical analysis was performed to compare baseline scores with end-of-study scores. However, HAM-d scores do appear lower for both groups at six-month follow-up (10.4–13.5) compared to baseline scores (21.8–22.2). **Alcohol use disorder** No differences in AUDIT scores were detected between the CBT and T-CBT group. However, both groups had a significantly lower AUDIT total score from baseline to end of treatment. The 13 participants who did not complete the AUDIT score at end-of-treatment were younger and more likely to meet remission criteria on the HAM-d score than paticipants completing the AUDIT.
Felleman et al.	An observational study including primary care patients (n = 224) identified with depression and with either no SUD (n = 98) or concurrent SUD (n = 126). Patients were recruited from three integrated primary care clinics in the Pacific Nothwest implementing a Primary Care Behavioral Health model. Patients were randomly selected based on their medical record. Patient exclusion criteria: Not attending a primary care appointment in the previous year; and records missing a depression score. The exact study period is unclear. Patients were followed for 12 months after enrollment.	**Substance use** Identified in the electronic medical record with a diagnosis established through clinical interviews, where the clinician was informed of endorsement of substance-specific items in the Global Appraisal of Individual Needs - Short Screener (GSS). **Depression** Depression symptoms were assessed using the Patient Health Questionnaire-9 (PHQ-9). The PHQ-9 score was dichotomized into low (0–9) and high depression (10–27).	**Primary Care Behavioral Health** **(PCBH) model** Integration of psychological and medical treatment in a primary care setting. PCBH is described as more comprehensive than a co-located mode, since primary care and mental health services are integrated within the primary care clinic. This entails that the behavioural health specialist is integrated into the treatment process. In the three primary care sites implementing the model, all primary care patients were screened for mental health disorders and referred to a behavioural health counsellor if the screening indicated presence of a a mental health disorder.	**Behavioral health services** **utilization (BHS)** Number of appointments with the primary care facilities’ behavioral health counselors within a 12-month period. **Medical treatment utilization (MTU)** Number of primary care appointments with medical providers within a 12-month period. Phone consultations, offsite in-patient hospitalizations, and medication check-ups were not included.	**Behavioral health services** **utilization (BHS)** Patients with a co-occurring diagnosis of SUD and severe depression had a mean number of 3.88 (SD 3.14) BHS appointments within the 12 months of follow-up. These patients did not have significantly more appointments compared to patients without SUD. **Medical treatment utilization (MTU)** Patients with a co-occurring diagnosis of substance use disorder and high depression had a mean number of 2.13 (SD 2.04) MTU appointments within the 12 months of follow-up. These patients did not have significantly more appointments compared to patients without SUD. **Moderating effect of SUD and depression on BHS** Patients with SUD tended to have a higher use of BHS if their depression level was high, however not significant. **Moderating effect of SUD and depression MTU through BHS** Patients with co-occuring SUD and depression had reduced use of primary care, presumeably due to an elevated use of BHS services.
Padwa et al.	An implementation study of Project Care from three primary care clinic organizations located in California. A total of nine clinic sites were included in the analytic sample. The study was conducted between 2011 and 2013. Follow-up site visits were conducted every 11–18 months and qualitative data collection was carried out throughout the study period.	**Mental health conditions** **Subtance use conditions** Details on diagnosis definition not further specified in the study.	**Project Care** Consisted of: Onsite mental health and substance use screenings for primary care patients; onsite brief intervention and treatment services for patients with mild to moderate mental health and/or subtance use conditions; referrals to specialty mental health and substance use services for individuals with more chronic or acute conditions; regular case management meetings; the use of electronic health records and registries; provider training about integrated care; and reimbursement for behavioral health services delivered on the same day as medical care visits, which are not currently covered by California’s Medicaid program.	**Capacity to deliver integrated** **services** The Behavioral Health Integration in Medical Care (BHIMC) tool was used to assess Clinic site’s capacity to deliver integrated primary care and behavioural health services. **Qualitative data** Addressing contextual factors that influence implementation.	**Implementation of integrated care in primary care clinics** Factors promoting or inhibiting the development of an integrated care capacity were related to outer- and inner contextual factors. Over time, clinics succeeded in increasing integrated care if they had support such as evaluation training and technical assistance services. It was hard to provide fully integrated behavioral health services and most difficulties were according to enhancing capacities related to substance use medications. Conclusion: both inner and outer contextual factors may impact this development in primary care clinics.
Urada et al.	Survey investigating perceptions of integrated treatment including primary care employees at nine sites. The following employee groups included (1) MH/SUD staff, (2) support staff (SS; medical assistants, administrative assistants, front office staff, and medical records staff), and (3) primary care providers (PCPs; nurses, physicians, physicians assistants). For study length and follow-up see "Padwa et al."	**Mental health conditions** **Subtance use conditions** Details on diagnosis definition not further specified in the study.	**Project Care** See description under "Padwa et al."	**Employees perceptions and satisfaction of integrated mental health and substance use treatment in a primary care setting** Survey included questions regarding: effectiveness and comfort, value of integration, and communication.	**Employee perceptions** Integration of MH/SUD services in to primary care was highly valued by staff and they were interested in further MH/SUD training (all types of staff). **Detected challenges** There were some problems according to information that seemed to flow better from MH/SUD staff to PCPs than in the other direction.

### Description of interventions

Overall, the identified studies can be divided into interventions testing (1) integrated care of mental disorders, substance use, and/or primary care [25–30], or (2) delivery of cognitive behavioral therapy (CBT) [31]. The integrated care models differ somewhat in the locus of the intervention. Park et al. [29] tested a Chronic Care management model with a specific focus on substance dependance treatment compared to usual primary care. The PRISM-E study (Bartels and Oslin et al. [25, 27]) had co-located mental- and substance use treatment which was compared to enhanced referral care offering similar treatment in a separate location. The Primary Care Behavioral Health (PCBH) model was tested by Felleman et al. [26] and entailed mental health services embedded within the primary care clinic. “Project Care” (Padwa and Urada et al. [28, 30]) introduced onsite screenings, treatment, referral, and case management of mental and substance use disorders. One study did not test integrated care but different ways of delivering CBT either face-to-face or telephone administered [31].

### Investigated outcomes and findings

The outcomes used for assessing the interventions can broadly be categorized into four different areas related to (1) mental health assessment, (2) substance and/or alcohol use assessment, (3) healthcare utilization or treatment engagement, and (4) implementation of the intervention.

We divided outcomes related to mental health into assessments regarding either changes in depression- or anxiety levels. To assess changes in depression level the studies used the patient health questionnaire (PHQ-9) [29, 31] and the Hamilton Depression Rating Scale (HAM-D) [31] as psychometrics. Receiving antidepressants and meeting criteria for major depression were also used to assess changes in depression level. Anxiety severity was measured by using the Beck Anxiety Inventory (BAI) [29]. These outcomes were used to assess an integrated care model (CCM) [29] versus usual primary care and face-to-face CBT [31] versus telephone administered CBT. None of the studies yielded significant differences in patients with dual diagnosis.

The Addiction Severity Index (ASI) [29], the Short Inventory of Problems for Alcohol and Drug Use (SIP) [29], the Alcohol Use Disorder Identification Test (AUDIT) [31], and quantity and frequency of alcohol consumption [27], were used to assess changes in substance- and alcohol use. No significant differences for any outcome were found for integrated care interventions on substance or alcohol use for patients with dual diagnosis. Looking at CBT, Kalatapapu et al. [31] did not find a significant difference between the groups having either face-to-face or telephone delivered CBT, however a significant decrease in alcohol use from baseline to end of study was detectable in both groups. It should however be mentioned, that around 25% of the study population dropped out of treatment, and a sub-analysis revealed that these individuals were more likely to meet the criteria for depression remission [31].

Healthcare utilization was assessed by either a count number of contact points with different healthcare facilities [26, 29], attendance as showing up for a scheduled appointment, or treatment drop-out [25, 26, 31]. None of the studies showed any significant differences in outcomes regarding healthcare utilization. The CBT study by Kalapatapu et al. [31] showed a discontinuation of treatment for 25% of patients, who were more prone to fulfill remission criteria for depression.

Implementation outcomes were reported in relation to Project Care (which was investigated in two of the included studies) [28, 30]. Regarding the integrated model in Project Care, The Behavioral Health Integration in Medical Care (BHIMC) tool was used to assess the capacity to deliver integrated services, employee’s perceptions of the model were assessed in a survey, and moreover contextual factors which impacted implementation were assessed qualitatively. Overall, Project Care was well received by employees, but some issues concerning the flow of information in the integrated model (mostly from primary care to behavioral health services) were identified, which yielded some difficulties in implementing an integrated model in a primary care setting.

### Target population

Scoping the literature, we detected major differences in the definitions of dual diagnosis in the identified studies, and no interventions were specifically targeted patients with dual diagnosis in our understanding as co-occurring SMI (severe depression, psychotic or bipolar disorders) and SUD.

Regarding the definition of SMI, three of the identified interventions were tested primarily on patients with depression [26, 29, 31]. The study by Park et al. furthermore included patients with PTSD [29]. In the remaining studies mental health disorders were defined in broader terms; Mental health conditions and Mental health problems respectively [25, 27, 28, 30].

The identified studies also used different definitions of SUD. SUD was defined either by using screening tools, having a diagnosis with SUD in a medical record, or by assessing the regularity or amount of alcohol or drug consumption. The studies by Bartels et al., Oslin et al. and Kalapatu et al. only included patients with problematic or at-risk alcohol consumption [25, 27, 31]. SUD in Felleman et al., Padwa et al. and Urada et al. were described as “substance use conditions” however, these were not specified further [26, 28, 30].

## Discussion

In this article, we have performed a scoping review of the literature investigating interventions in general practice targeting patients with dual diagnosis. We included seven studies in our narrative synthesis. The identified interventions consisted primarily of different types of integrated treatment models in various organization modes and combinations plus one study focusing on the delivery of CBT treatment. A range of different outcomes was used to evaluate the interventions, where psychometrics primarily were used for assessing depression and anxiety levels, and consumption measures were used to assess SUD. No significant differences were found in mental health or SUD improvements. Furthermore, two of the identified studies evaluated intervention implementation of an integrated care model and found implementation feasible, however, the information flow from primary care to behavioural health services could be improved.

From our analysis of the literature regarding interventions targeting patients with dual diagnosis in a general practice context, we have identified a tremendous gap. There could be several reasons for this. Of the seven studies we identified, none evaluated interventions that directly targeted patients with SMI and co-occurring SUD. The primary mental disorder was major depression, and we found no studies that included either affective bipolar disorder or psychotic disorders. Moreover, the most common SUD were alcohol use disorder or at-risk drinking. Only one study included drug use in their definition of SUD. This lack of interventions targeting populations with more severe disorders could be a result of our focus on the general practice setting. One could argue that general practice does not carry the primary treatment responsibility for this patient population – particularly not the patients who are severely mentally ill and are concurrently using substances actively. These patients are, in most parts (in most countries), treated and tended to in the psychiatric system, who also diagnose SMI, and prescribe- and regulate psychotropic drug prescriptions. This might also be the underlying reason that no pharmaceutical treatment interventions were identified in the review, since medication prescriptions related to the treatment of SMI often is prescribed and administered in the secondary healthcare sector. However, general practitioners (GPs) do play a pivotal role, when it comes to identifying risk factors in the initial stages of disease development (including identification of mental illnesses and substance use disorders) [32] and coordinating care across healthcare sectors, and continuity of care with a regular GP have been found to lower acute hospital admissions and mortality in patients in general [33]. Since this population is particularly vulnerable towards chronic somatic comorbidities, the findings from our study could call for novel research interventions on dual diagnosis in general practice in other contexts.

### Limitations

The purpose of the study was to identify interventions evaluated in a general practice setting. Since we only ended up including seven studies, it might be relevant to scope the broader literature by including other study designs. By including a more explorative study design, we could potentially gain a deeper understanding of the landscape of dual diagnosis within general practice. We only included studies published in English, and thus we might have overlooked findings from publications outside the Global North. Moreover, the definition of dual diagnosis has been discussed in the literature. The concept of dual diagnosis was introduced in the 1980s [4], but today there is no common diagnostic criterion for the condition. Hence, there may be terms for dual diagnosis that were not covered in our search string, cf. appendix.

Contrary to the concept of dual diagnoses, there are many well-defined terms for general practice, however, large differences exist globally regarding healthcare structures. Surprisingly, all interventions included for analysis were situated in the United States. Since the US has a unique healthcare system based on insurance and, at large, self-payment structures, transferring the results to other healthcare systems organized differently could prove to be difficult. Lastly, our definition of interventions in the study is somewhat narrow. We only included interventions tested or evaluated, however we could have broadened the scope of the study to include study protocols, programs, initiatives, or treatments. This might have contributed to a broader perspective on the landscape of general practice and dual diagnoses. Overall, this has several implications, that one may have both overlooked and included articles that should not have been included in the review, as well as potentially not having a complete picture of the interventions that are in the field.

### Conclusion

Only seven intervention studies targeting patients with dual diagnosis in the setting of general practice were found in this scoping review. This finding could suggest a lack of research within this area and points towards a potential for future investigations and testing of interventions targeting this vulnerable patient population in a general practice setting.

### Electronic supplementary material

Below is the link to the electronic supplementary material.

Supplementary Material 1

## Data Availability

N/A.

## Abbreviations

SMI: Severe mental illness
SUD: Substance Use Disorder
CBT: Cognitive Behavioral Treatment
GP/GPs: General practitioner/General practitioners

## References

[1] Chang CK, Hayes RD, Perera G, et al. Life expectancy at birth for people with serious mental illness and other major disorders from a secondary mental health care case register in London. PLoS ONE. 2011;6(5):e19590. 10.1371/journal.pone.0019590. PubMed PMID: 21611123; PubMed Central PMCID: PMCPMC3097201. eng. 21611123 10.1371/journal.pone.0019590 PMC3097201

[2] Hjorthøj C, Østergaard ML, Benros ME, et al. Association between alcohol and substance use disorders and all-cause and cause-specific mortality in schizophrenia, bipolar disorder, and unipolar depression: a nationwide, prospective, register-based study. Lancet Psychiatry. 2015;2(9):801–8. 10.1016/s2215-0366(15)00207-2. PubMed PMID: 26277044; eng. 26277044 10.1016/s2215-0366(15)00207-2

[3] Nordentoft M, Wahlbeck K, Hällgren J, et al. Excess mortality, causes of death and life expectancy in 270,770 patients with recent onset of mental disorders in Denmark, Finland and Sweden. PLoS ONE. 2013;8(1):e55176. 10.1371/journal.pone.0055176. PubMed PMID: 23372832; PubMed Central PMCID: PMCPMC3555866. eng. 23372832 10.1371/journal.pone.0055176 PMC3555866

[4] Hryb K, Kirkhart R, Talbert R. A call for standardized definition of dual diagnosis. Psychiatry. 2007;4:15–6. 20532112 PMC2880934

[5] Toftdahl NG, Nordentoft M, Hjorthøj C. Prevalence of substance use disorders in psychiatric patients: a nationwide Danish population-based study. Soc Psychiatry Psychiatr Epidemiol. 2016;51(1):129–40. 10.1007/s00127-015-1104-4. PubMed PMID: 26260950; eng. 26260950 10.1007/s00127-015-1104-4

[6] Volkow ND. Substance use disorders in schizophrenia–clinical implications of comorbidity. Schizophr Bull. 2009;35(3):469–72. 10.1093/schbul/sbp016. PubMed PMID: 19325163; PubMed Central PMCID: PMCPMC2669586. 19325163 10.1093/schbul/sbp016 PMC2669586

[7] Pourat N, Chen X, Lee C, et al. Improving Outcomes of Care for HRSA-Funded Health Center patients who have Mental Health conditions and Substance Use disorders. J Behav Health Serv Res. 2020;47(2):168–88. PubMed PMID: 31214934. 31214934 10.1007/s11414-019-09665-5

[8] Stoskopf CH, Kim YK, Glover SH. Dual diagnosis: HIV and mental illness, a population-based study. Community Ment Health J. 2001;37(6):469–79. 10.1023/a:1017577827658. PubMed PMID: 11504140; eng. 11504140 10.1023/a:1017577827658

[9] Roncero C, Buch-Vicente B, Martín-Sánchez ÁM, et al. Prevalence of hepatitis C virus infection in patients with chronic mental disorders: the relevance of dual disorders. Gastroenterol Hepatol. 2022;30. 10.1016/j.gastrohep.2022.06.005. PubMed PMID: 35780956; eng spa. 10.1016/j.gastrohep.2022.06.005 35780956

[10] Chesher NJ, Bousman CA, Gale M, et al. Chronic illness histories of adults entering treatment for co-occurring substance abuse and other mental health disorders. Am J Addict. 2012;21(1):1–4. 10.1111/j.1521-0391.2011.00196.x. PubMed PMID: 22211340; PubMed Central PMCID: PMCPMC3629909. 10.1111/j.1521-0391.2011.00196.x PMC3629909 22211340

[11] Hannerz H, Borgå P, Borritz M. Life expectancies for individuals with psychiatric diagnoses. Public Health. 2001;115(5):328–37. 10.1038/sj.ph.1900785. PubMed PMID: 11593442; eng. 11593442 10.1038/sj.ph.1900785

[12] Laursen TM, Munk-Olsen T, Gasse C. Chronic somatic comorbidity and excess mortality due to natural causes in persons with schizophrenia or bipolar affective disorder. PLoS ONE. 2011;6(9):e24597. 10.1371/journal.pone.0024597. PubMed PMID: 21935426; PubMed Central PMCID: PMCPMC3173467. eng. 21935426 10.1371/journal.pone.0024597 PMC3173467

[13] Correll MDEH, Bobes CU. Physical illness in patients with severe mental disorders. I. Prevalence, impact of medications and disparities in health care. World Psychiatry. 2011;10(1):52–77. 10.1002/j.2051-5545.2011.tb00014. x. PubMed PMID: 21379357; PubMed Central PMCID: PMCPMC3048500. eng. 21379357 10.1002/j.2051-5545.2011.tb00014 PMC3048500

[14] Vancampfort D, Stubbs B, Mitchell AJ, et al. Risk of metabolic syndrome and its components in people with schizophrenia and related psychotic disorders, bipolar disorder and major depressive disorder: a systematic review and meta-analysis. World Psychiatry. 2015;14(3):339–47. 10.1002/wps.20252. PubMed PMID: 26407790; PubMed Central PMCID: PMCPMC4592657. eng. 26407790 10.1002/wps.20252 PMC4592657

[15] Gardner-Sood P, Lally J, Smith S, et al. Cardiovascular risk factors and metabolic syndrome in people with established psychotic illnesses: baseline data from the IMPaCT randomized controlled trial. Psychol Med. 2015;45(12):2619–29. 10.1017/s0033291715000562. PubMed PMID: 25961431; PubMed Central PMCID: PMCPMC4531468. eng. 25961431 10.1017/s0033291715000562 PMC4531468

[16] Lawrence D, Kisely S. Inequalities in healthcare provision for people with severe mental illness. J Psychopharmacol. 2010;24(4 Suppl):61–8. 10.1177/1359786810382058. PubMed PMID: 20923921; PubMed Central PMCID: PMCPMC2951586. eng. 20923921 10.1177/1359786810382058 PMC2951586

[17] Lawrence R. Primary care and dual diagnosis. In: GH R, editor. Dual diagnosis nursing. Malden: Blackwell Publishing; 2006. pp. 140–9.

[18] Thylstrup B, Johansen KS, Sønderby L. Treatment effect and recovery — dilemmas in dual diagnosis treatment. Nordic Stud Alcohol Drugs. 2017;26(6):552–60. 10.1177/145507250902600601. 10.1177/145507250902600601

[19] Kêdoté MN, Brousselle A, Champagne F. Use of health care services by patients with co-occurring severe mental illness and substance use disorders. Ment Health Subst Use. 2008;1(3):216–27. 10.1080/17523280802274886. PubMed PMID: 27239226; PubMed Central PMCID: PMCPMC4882163. eng. 27239226 10.1080/17523280802274886 PMC4882163

[20] Johansen KS. Treatment of dual diagnosis in Denmark. Qualitative Stud. 2018;5(2):125–39. 10.7146/qs.v5i2.104500. 10.7146/qs.v5i2.104500

[21] Arksey H, O’Malley L. Scoping studies: towards a methodological framework. Int J Soc Res Methodol. 2005 2005/02/01;8(1):19–32. 10.1080/1364557032000119616.

[22] Alsuhaibani R, Smith DC, Lowrie R, et al. Scope, quality and inclusivity of international clinical guidelines on mental health and substance abuse in relation to dual diagnosis, social and community outcomes: a systematic review. BMC Psychiatry. 2021;21(1):209. 10.1186/s12888-021-03188-0. PubMed PMID: 33892659; PubMed Central PMCID: PMCPMC8066498. 33892659 10.1186/s12888-021-03188-0 PMC8066498

[23] Team TE. EndNote [64 bit]. EndNote X9. Philadelphia, PA: Clarivate; 2013.

[24] Innovation VH. Covidence systematic review software Melbourne, Australia. Available from: www.covidence.org.

[25] Bartels SJ, Coakley EH, Zubritsky C, et al. Improving access to geriatric mental health services: a randomized trial comparing treatment engagement with integrated versus enhanced referral care for depression, anxiety, and at-risk alcohol use. Am J Psychiatry. 2004;161(8):1455–62. 10.1176/appi.ajp.161.8.1455. PubMed PMID: 15285973; eng. 15285973 10.1176/appi.ajp.161.8.1455

[26] Felleman BI, Athenour DR, Ta MT et al. Behavioral health services influence medical treatment utilization among primary care patients with comorbid substance use and depression. J Clin Psychol Med Settings. 2013;20(4):415 – 26. 10.1007/s10880-013-9367-y. PubMed PMID: 23744107; eng. 10.1007/s10880-013-9367-y 23744107

[27] Oslin DW, Grantham S, Coakley E, et al. PRISM-E: comparison of integrated care and enhanced specialty referral in managing at-risk alcohol use. Psychiatr Serv. 2006;57(7):954–8. 10.1176/ps.2006.57.7.954. PubMed PMID: 16816279; PubMed Central PMCID: PMCPMC3169203. eng. 16816279 10.1176/ps.2006.57.7.954 PMC3169203

[28] Padwa H, Teruya C, Tran E, et al. The Implementation of Integrated Behavioral Health protocols in primary care settings in Project Care. J Subst Abuse Treat. 2016;62:74–83. PubMed PMID: 26683125; eng. 26683125 10.1016/j.jsat.2015.10.002

[29] Park TW, Cheng DM, Samet JH, et al. Chronic care management for substance dependence in primary care among patients with co-occurring disorders. Psychiatr Serv. 2015;66(1):72–9. 10.1176/appi.ps.201300414. PubMed PMID: 25219686; PubMed Central PMCID: PMCPMC4282827. eng. 25219686 10.1176/appi.ps.201300414 PMC4282827

[30] Urada D, Schaper E, Alvarez L et al. Perceptions of mental health and substance use disorder services integration among the workforce in primary care settings. J Psychoact Drugs 2012 Sep-Oct;44(4):292–8. 10.1080/02791072.2012.720163. PubMed PMID: 23210377; eng. 10.1080/02791072.2012.720163 23210377

[31] Kalapatapu RK, Ho J, Cai X et al. Cognitive-behavioral therapy in depressed primary care patients with co-occurring problematic alcohol use: effect of telephone-administered vs. face-to-face treatment-a secondary analysis. J Psychoact Drugs 2014;46(2):85–92. 10.1080/02791072.2013.876521. PubMed PMID: 25052784; PubMed Central PMCID: PMCPMC4110640. eng. 10.1080/02791072.2013.876521 PMC4110640 25052784

[32] Marshall KL, Deane FP. General practitioners’ detection and management of patients with a dual diagnosis: Implications for education and training. 2004;23(4):455–62. 10.1080/09595230412331324572. 10.1080/09595230412331324572 15763750

[33] Sandvik H, Hetlevik Ø, Blinkenberg J, et al. Continuity in general practice as predictor of mortality, acute hospitalisation, and use of out-of-hours care: a registry-based observational study in Norway. Br J Gen Pract. 2022;72(715):e84–90. 10.3399/bjgp.2021.0340. PubMed PMID: 34607797; PubMed Central PMCID: PMCPMC8510690. eng. 34607797 10.3399/bjgp.2021.0340 PMC8510690

