# Supplementary material for: Interventions targeting patients with co-occuring severe mental illness and substance use (dual diagnosis) in general practice settings – a scoping review of the literature
Source: BMC Prim Care. 2024 Aug 3;25:281. doi: 10.1186/s12875-024-02504-3 (PMC11297724; doi:10.1186/s12875-024-02504-3)
Supplement: Supplementary file 1 — Supplementary Material 1 [file 12875_2024_2504_MOESM1_ESM.docx]

**Appendix 1**

A combination of MeSH terms, keywords, free text search, as well as related terms, were used with Boolean operators (AND, OR). Each search was adapted to the specific requirements of the individual database, where the search string was divided into blocks of resp. dual diagnosis (Block 1), general practice (block 2), and intervention (block 3). The final search string was as follows:

- ("Diagnosis, Dual (Psychiatry)" [MESH] OR "Dual Diagnosis" OR "Concurrent disorder*" OR "Substance use disorder*" OR ("Substance abuse*" AND ("Mental illness" OR "Mental disorder"))) AND ("Family Practice" [MESH] OR "General Practice" [MESH] OR " Primary health care" [MESH] OR "General Practice" OR "Primary health care" OR "Family Practice") AND ("Mental Health Services"[Mesh] OR Intervention* OR Treatment* OR Initiative* OR Utilization* OR Therapy)
